# Editorial: Sphingolipid metabolism dysfunction in cancer

**DOI:** 10.3389/fendo.2023.1208616

**Published:** 2023-06-09

**Authors:** Rashika El Ridi, Jingjing Duan, Junfei Jin

**Affiliations:** ^1^ Zoology Department, Faculty of Science, Cairo University, Giza, Egypt; ^2^ Human Aging Research Institute and School of Life Sciences, Nanchang University, Nanchang, China; ^3^ Guangxi Key Laboratory of Molecular Medicine in Liver Injury and Repair, Guangxi Health Commission Key Laboratory of Basic Research in Sphingolipid Metabolism Related Diseases, Affiliated Hospital of Guilin Medical University, Guilin, China

**Keywords:** sphingolipid, metabolism, cancer, mechanism, endocrine disorder

Sphingolipids are important structural components of biological membranes, maintaining the barrier function and fluidity of cell membranes. Sphingolipids have been shown to modulate the expression, accessibility, and activity of surface receptors on tumor and immune cell surfaces. Additionally, sphingolipids are bioactive molecules that play a predominant role in signal transduction, cell growth, differentiation, senescence, and programmed cell death and thus regulate cancer cell signaling and affect tumor suppression or survival. In support of these observations, Hu et al. reported that hyperactivation of sphingolipid metabolism was associated with upregulation of Notch signaling and the angiogenesis pathway, contributing to metastasis and poor prognosis. Furthermore, changes in the expression of multiple immune genes indicated that sphingolipid metabolism might disrupt tumor biological processes and alter the immune status. Zhong et al. identified vital sphingomyelin metabolism-related genes in osteosarcoma using 88 frozen samples and established their impact on patient prognosis, progression, and survival via statistical analyses and a risk-based scoring model. The results of Hu et al. and Zhong et al. shed new light on the molecular mechanisms involving sphingolipid metabolism in osteosarcoma, which could provide valuable information for the development of targeted therapeutics for this disease.

The research article of Yuan et al. provided further evidence of the critical role of sphingolipid metabolism in cancer development and highlighted the potential for targeting sphingolipid metabolism as a therapeutic strategy in colon adenocarcinoma. Univariate Cox regression analysis suggested that 26 sphingolipid metabolism-related genes could be used to predict prognosis, and a majority of these genes might serve as risk genes for this cancer, implying a potential pathogenic effect of these genes on cancer development. In addition, the influence of sphingolipid metabolism on the tumor immune milieu was investigated further, laying the groundwork for the investigation of the link between sphingolipid metabolic changes and the tumor immune microenvironment. The authors were able to develop a unique and reliable prognostic discriminant model based on the sphingolipid metabolism classification technique.

The study by Chi et al. capitalized on documented information implicating aberrant sphingolipid metabolism in the initiation, development, diagnosis and prognosis of uveal melanoma, the most common primary and metastatic intraocular malignancy in adults. Their research used machine learning to establish the sphingolipid gene expression status and its impact on the tumor and the immune microenvironment. The researchers analyzed the level of immune infiltration in high- and low-risk patients using platforms such as CIBERSORT and found that twenty-seven sphingolipid metabolism-related genes were associated with malignancy. A signature comprising four sphingolipid metabolism genes was strongly associated with immune checkpoint expression and the immune microenvironment in uveal melanoma, and genes controlling immune checkpoint expression were found to characterize the immune landscape and were strongly associated with the prognosis of this ocular cancer. Chi et al. also assessed the predictive value of the four sphingolipid metabolism genes in the response to immunotherapy and immune checkpoint blockade (ICB) using the ImmuCellAI and TIP portals.

The aforementioned different studies thus essentially focused on sphingolipid metabolism genes and provided an invaluable platform for sorely needed biochemical investigations on the sphingolipid species distribution and content in different tumors. However, more studies are needed to answer some key questions and elucidate detailed mechanisms related to sphingolipid metabolism dysfunction in cancer.

## Author contributions

RE drafted the manuscript, JD revised it, and JJ finalized it. All authors contributed to the article and approved the submitted version.

